# Supplementary material for: Silencing miR-146a-5p Protects against Injury-Induced Osteoarthritis in Mice
Source: Biomolecules. 2023 Jan 7;13(1):123. doi: 10.3390/biom13010123 (PMC9856058; doi:10.3390/biom13010123)

Supplemental Table S1. Primer sequences for real-time qPCR analyses

Genes	Sequences
Il6	5′-TCC AGT TGC CTT CTT GGG AC-3′
	5′-GTA CTC CAG AAG ACC AGA GG-3′
Tnfa	5′-CAC ACT CAG ATC ATC TTC TCA A-3′
	5′-AGT AGA CAA GGT ACA ACC CAT C-3′
Mmp13	5'-AGA CTG GTA ATG GCA TCA AGG-3'
	5'-GCC ATT TCA TGC TTC CTG ATG-3'
Actb	5’-AGA TGT GGA TCA GCA AGC AG-3’
	5’-GCG CAA GTT AGG TTT TGT CA-3’
IL6	5’-GCC TTC GGT CCA GTT GCC TT-3’
	5’-GCA GAA TGA GAT GAG TTG TC-3’
TNFa	5’-AGG CGC TCC CCA AGA AGA CA-3’
	5’-TCC TTG GCA AAA CTG CAC CT-3’
MMP13	5’-TGG CAT CAA GGG ATA AGG AAG-3’
	5’-AGC CAC TTT ATG CTT CCT GA-3’
ACTB	5'-CCT TGC ACA TGC CGG AG-3'
	5'-ACA GAG CCT CGC CTT TG-3'

Supplemental Figure S1. Data analyses of miRNA-Seq assays with IL-1β or vehicle treated primary murine articular chondrocytes. (a) Principle component analysis (PCA) plot of two treatment groups (IL-1β or vehicle) (n=4). (b) Heatmap of correlation within samples (n=4 for each group).

Supplemental Figure S2. Knockdown efficacy of *miR-146a-5p* inhibitor in articular chondrocytes. Real-time qPCR for *miR-146a-5p* in primary murine articular chondrocytes transfected with different concentrations of *miR-146a-5p* inhibitor. $^*P < 0.05$ determined by two-way ANOVA followed by Tukey test to compare among multiple groups.

Supplemental Figure S3. Loss of *miR-146a* in chondrocytes does not lead to phenotypic abnormalities within joints. (a) Safranin O/fast green staining of uninjured contralateral knee sections of control sham and *miR-146a* LOF sham mice at 10 weeks post MLI (n=7) (scale bar, 100 μm). (b) MicroCT images of uninjured contralateral subchondral bone from control sham and *miR-146a* LOF sham mice at 10 weeks post MLI surgery (n=7) (scale bar, 0.5 mm). (c) BV/TV measured on uninjured contralateral subchondral bone from control sham and *miR-146a* LOF sham mice at 10 weeks post MLI surgery (n=7). Data presented as mean ± SD. $^*P < 0.05$ by Student's t test.

Supplemental Figure S4. Data analyses of RNA-Seq assays with IL-1β or vehicle treated primary murine articular chondrocytes. (a) PCA plot of two treatment groups (IL-1β or vehicle) (n=4). (b) Heatmap of correlation within samples (n=4 for each group).

Supplemental Figure S1

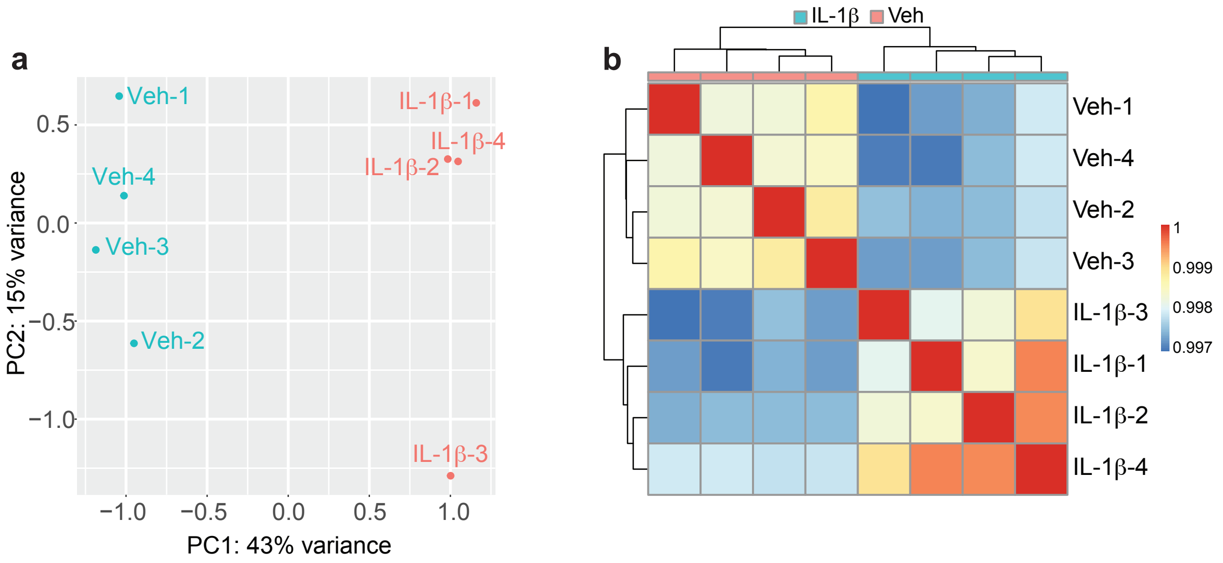

Supplemental Figure S2

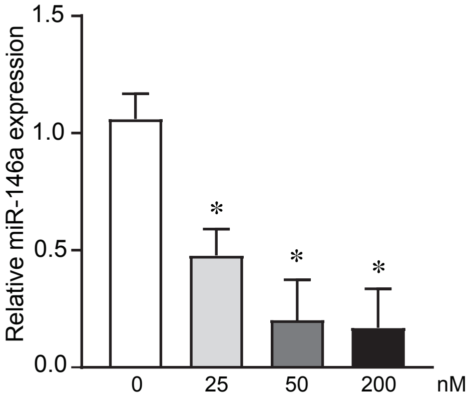

Supplemental Figure S3

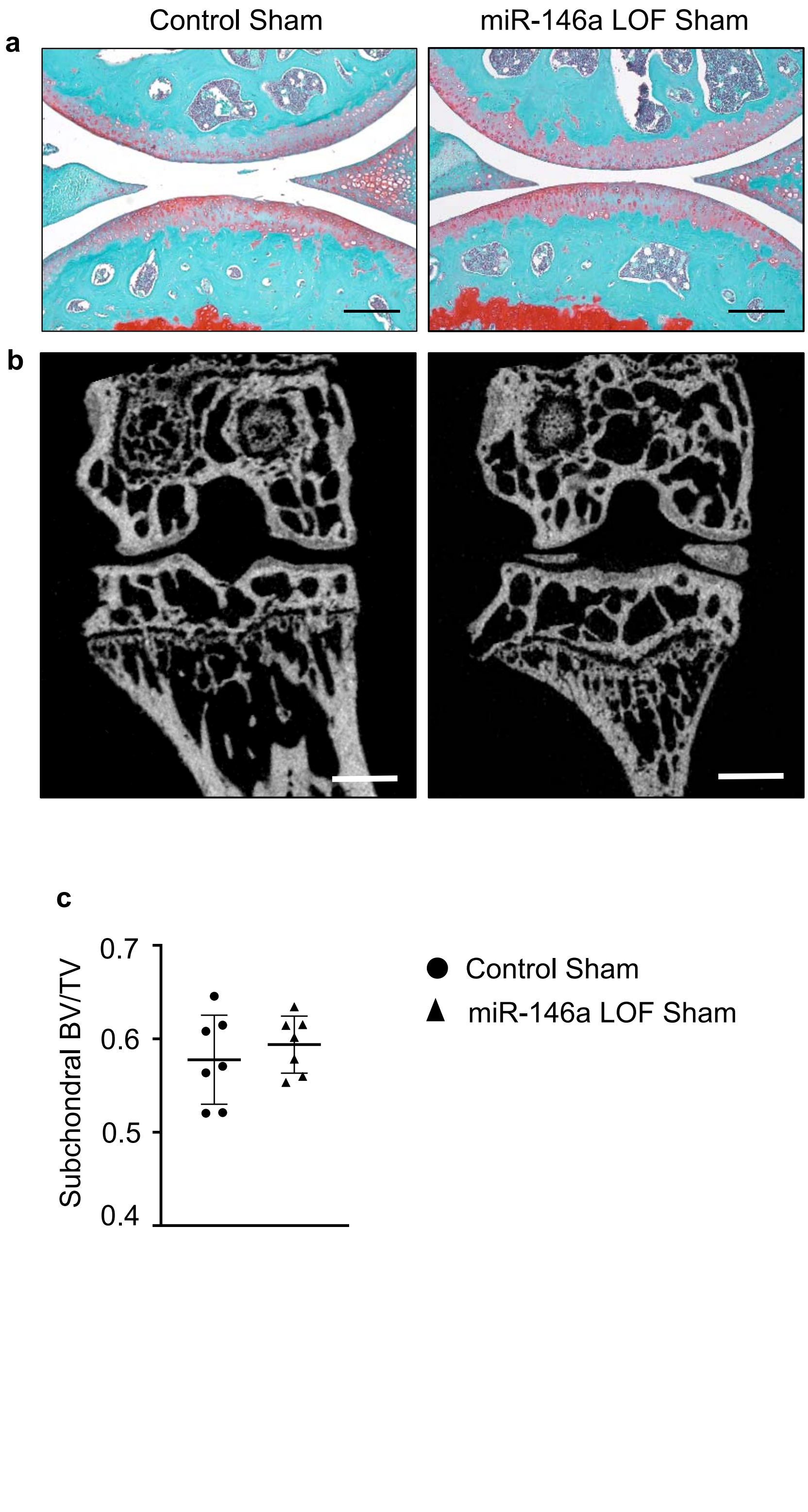

Supplemental Figure S4

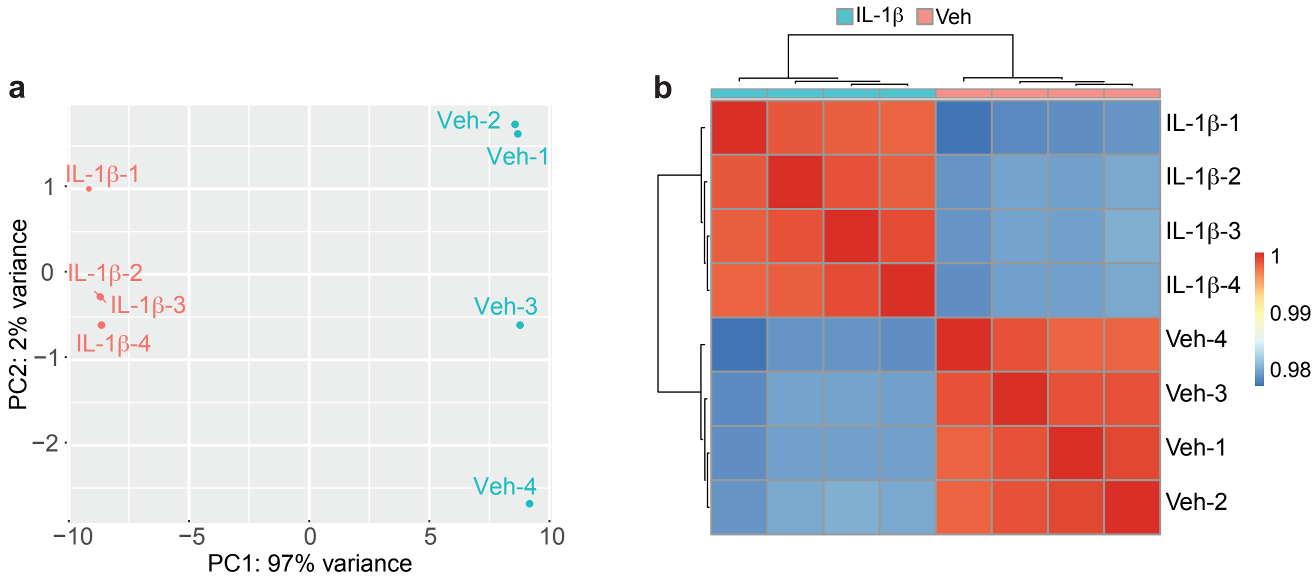

Supplement: Supplementary file 1 [file biomolecules-13-00123-s001.zip › biomolecules-2136633-supplementary.pdf]
